# An Allosteric Modulator of the Adenosine A_1_ Receptor Improves Cardiac Function Following Ischaemia in Murine Isolated Hearts

**DOI:** 10.3390/ph6040546

**Published:** 2013-04-12

**Authors:** Anna Butcher, Peter J. Scammells, Paul J. White, Shane M. Devine, Roselyn B. Rose’Meyer

**Affiliations:** 1 School of Medical Sciences, Griffith University, Gold Coast Campus, Southport, Queensland 4222, Australia; E-Mail: anna.butcher@griffithuni.edu.au; 2 Monash Institute of Pharmaceutical Sciences, Monash University, Parkville, Victoria 3052, Australia; E-Mails: peter.scammells@monash.edu (P.J.S.); paul.white@monash.edu (P.J.W.); Shane.Devine@monash.edu (S.M.D.)

**Keywords:** allosteric modulator, adenosine A_1_ receptor, cardioprotection, heart

## Abstract

The effect of an allosteric modulator of the adenosine A_1_ receptors was investigated using an ischaemia-reperfusion protocol in murine isolated hearts. Isolated hearts were perfused with Kreb-Henseleit solution gassed with carbogen gas (95% O_2_ and 5% CO_2_) in Langendorff mode and electrically paced at 480 bpm. Following 20 min equilibration and 20 min global normothermic ischaemia, the allosteric modulator VCP333 (1 μM) or the adenosine A_1_ receptor partial agonist VCP102 (10 μM) were infused after 5 min of reperfusion for 15 min. Upon termination of the drug treatment, reperfusion continued for a further 40 min. At the end of 60 min reperfusion, treatment with VCP333 or VCP102 improved the recovery of the left ventricular developed pressure when compared to control group responses (*p* < 0.05). Neither compound affected end diastolic pressure, coronary flow rates or dP/dt_max_ values when compared to control tissues during reperfusion (*p* > 0.05). The infusion of VCP102 or VCP333 during reperfusion reduced cardiac troponin I efflux to 6.7% and 25% respectively of control heart efflux (*p* < 0.05). This data indicates that the allosteric modulator of the adenosine A_1_ receptor (VCP333) has similar characteristics to the adenosine receptor partial agonist VCP102 as it improves cardiac function and reduces myocardial cell death following an ischaemic episode.

## 1. Introduction

Adenosine receptors belong to one of the largest families of cell surface proteins, the G-protein coupled receptors [1]. The four adenosine receptor subtypes (A_1_, A_2A_, A_2B_, A_3_) are found in the heart and are reported to regulate cardiac function [2,3], modulate coronary blood flow [4] and are cardioprotective [5].

When levels of metabolic oxygen decrease causing a reduction in cellular ATP levels, for example, in the case of myocardial ischaemia, adenosine production increases and is generated at regulated amounts depending upon the ratio of oxygen supply and demand. Adenosine that is released from myocardial cells then binds to its corresponding sarcolemmal membrane receptors [6]. The biological half-life of adenosine is approximately 0.6–1.5 s as it is recycled through membrane bound pumping systems back into cytosolic compartments [7]. All adenosine receptor subtypes play an important role in protection of the heart from ischaemia-reperfusion induced cell damage [8]. The adenosine A_1_ receptor is coupled to Gi/o to inhibit the formation of cAMP [1] and with respect to cardioprotection, the adenosine A_1_ receptor has been of particular focus to researchers in recent years. Extensive research involving transgenic mice have shown that overexpression of the adenosine A_1_ receptor is associated with a decrease in ischaemic damage to the heart [6]. It was reported that the increased expression of cardiac adenosine A_1_ receptors in the transgenic mouse models generated an ischaemic tolerant phenotype characterised by reduced contractile dysfunction, de-energisation, necrosis and infarction [9].

Full agonists with high efficacy bind to the adenosine A_1_ receptor, stimulate a full pharmacological response and includes compounds such as *N*^6^-cyclopentyladenosine (CPA, K_i_ 0.8 nM, and (R)-phenylisopropyladenosine (R-PIA; K_i_ 860 nM) [10]. In contrast, adenosine receptor partial agonists are low-efficacy ligands that compared to a full agonist, elicit only a sub-maximal response when occupying all (>95%) available receptors [11]. Both full and partial agonists of the adenosine A_1_ receptors have been proved to reduce ischaemic damage to the heart [12,13]. However, because adenosine and its receptors are widespread in the body, the targeting of adenosine receptor subtypes by selective full or partial agonists has many potential biological implications, as full agonists may induce desensitization of the receptors with prolonged exposure [14].

Similar to other G-protein coupled receptors, adenosine receptors show allosteric receptor sites topographically distinct from the classic orthosteric sites to which the previously studied adenosine A_1_ receptor agonists bind [15]. “Allosteric” comes from the Greek term meaning “other site”, and compounds acting at this site possess significant advantages over those acting at orthosteric sites. Firstly, the effects of allosteric enhancers are saturable; once the allosteric effects are completely occupied, no further effect is observed [16,17]. Secondly, they induce responses only in tissues in which endogenous agonists exert their physiological effects. Finally, they have a greater potential for receptor subtype selectivity [16]. Therefore, greater understanding into the mechanisms of action of these receptor target sites allows the potential for the development of novel drug tools and selective therapeutic agents.

The use of allosteric enhancers of the adenosine A_1_ receptors provides a strategy to target ischaemic tissues and isolate responses to adenosine. Adenosine A_1_ receptors are the first of the adenosine receptor subtypes for which allosteric modulators were described [15,18]. These receptors are classified as rhodopsin-like receptors (class A), where the orthosteric and allosteric sites are located within the cavity formed by the seven transmembrane domains [19]. During cardiac ischaemia-reperfusion injury, the concentrations of extracellular adenosine have been shown to rise over 100-fold over basal levels [12], providing a grounds for tissue specific and temporal selectivity of drug action targeting allosteric sites on adenosine receptors. Moreover, these receptor target sites have been investigated for their clinical use in paroxysmal supraventricular tachycardia [20].

**Figure 1:**
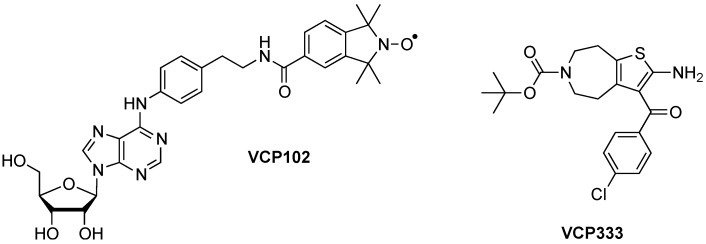
Chemical structures of VCP102 and VCP333.

A group of allosteric compounds, known as the 2-amino-3-benzoylthiophenes (2A3BTs) are an example of small-molecule allosteric modulators with agonist properties. These compounds have been shown to potentiate the effects of adenosine via the adenosine A_1_ receptor [15]. One compound in particular, *tert*-butyl 2-amino-3-(4-chlorobenzoyl)-7,8-dihydro-4*H*-thieno[2,3-*d*]azepine-6(5*H*)-carboxylate (VCP333) has been utilised in this study to determine its potential effects in preventing ischaemia-reperfusion injury in the isolated mouse heart. We compared VCP333 to the partial adenosine A_1_ receptor agonist VCP102, which we have previously observed to be cardioprotective in an ischaemia-reperfusion protocol in murine isolated hearts [13,21]. The chemical structures of VCP102 and VCP333 are shown in Figure 1.

The aim of the current study was analyse the effect of the allosteric modulator of the adenosine A_1_ receptor VCP333 infused post ischaemia on cardiac function in the mouse isolated heart preparation. We examined the effect of 15 min of VCP333 or VCP102 infusion during early reperfusion on selected functional parameters and on oncosis and necrosis.

## 2. Experimental

### 2.1. Perfused Heart Preparation

The Griffith University Animal Ethics Committee approved this project to use 6–10 mice per group. Animal numbers were kept to a minimum by ensuring that experimental protocols were strictly adhered to, to reduce variability in the data sets. Isolated heart preparations were performed using 6–7 week old male C57/Black mice, anaesthetised with 50 mg kg^−1^ sodium pentobarbitone IP. Sodium pentobarbitone was implemented to allow rapid anaesthesia prior to removal of hearts, which reduced damage to the preparation and reduce variability in the data. Hearts were rapidly excised and placed in ice-cold perfusion fluid. The aorta was cannulated (21-gauge modified stainless steel needle) and held in place with an aneurysm clip before being secured using a 4-0 silk suture. Hearts were perfused in a retrograde way at a hydrostatic pressure of 80mm Hg with a modified Krebs-Henseleit solution containing (in mM): NaCl 120; NaHCO_3_ 22; KCl 4.7; K_2_HPO_4_ 1.2; Glucose 11.0; MgCl_2_ 1.2; CaCl_2_ 2.5 and EDTA 0.5, which was prepared in Milli-Q water at room temperature.

The perfusing solution was equilibrated with carbogen gas (95% O_2_ and 5% CO_2_) heated to 37 °C. The physiological salt solution was passed through a 0.45 µM Sterivex-HV filter (Millipore, Bedford, MA, USA) in order to provide continuous removal of particulate matter. In order to avoid raised intraventricular pressure due to accumulation of perfused buffer, a small polyethylene tube was inserted through the left ventricle and then out through the apex of the heart.

A small fluid filled balloon of approximately 5 mm diameter was inserted via the mitral valve into the left ventricle to permit a continuous measurement of left ventricular function. Hearts were then placed into a glass organ bath which provided continuous immersion in the perfusate at 37 °C. The temperature of the physiological salt solution was constantly monitored using the Fluke 51 II Thermometer (Fluke Corporation, Everett, WA, USA), which was connected to a needle thermistor at the entry into the aortic cannula.

The Langendorff isolated heart perfusion system permitted the measurement of various functional parameters including coronary flow rate, left ventricular developed pressure (LVDP), left ventricular end diastolic pressure (EDP), contractility (measured through the inotropic changes, max/min dP/dt) and heart rate. Coronary flow rate was monitored using an ultrasonic flow probe connected to a 1-channel flow meter (Model T106, Transonic Systems, Ithaca, NY, USA). An end-diastolic pressure of approximately 5mmHg was achieved by inflating the fluid-filled ventricular balloon using a 500 µL Hamilton threaded plunger syringe (Hamilton Co., Reno, NV, USA).

After 15 min equilibration, hearts were paced at 9 Hz (0.5 m duration, supramaximal voltage) using a 611 Stimulator (Phipps and Bird Inc, Richmond, VA, USA). To commence normothermic ischaemia, coronary flow and pacing to the heart was then switched off for 20 min and maintained at 37 °C in the isolated bath. Data samples in all experiments were taken at pre-pacing, 10-min pre-ischaemia, 5-min intervals during ischaemia and at 1, 2, 5, 10 and at 10-min subsequent intervals during 60 min of reperfusion.

We assessed functional recovery in isolated mouse hearts subjected to 20 min of ischaemia followed by 60 min of reperfusion using the compound VCP333 at 1 µM (K_B_ 3.55 ± 0.1 μM, [15]) or VCP102 at 10 μM (K_i_ 7 ± 1 nM [13]). The experimental treatments were randomized into control or drug treated hearts. The concentration of VCP102 used in these experiments was demonstrated to be effective in isolated H9c2 cardiac myocyte experiments. The concentration of VCP333 utilised in these experiments was the level found to be effective in membrane-based and intact-cell radioligand binding, multiple signaling assays, and a native tissue bioassay [15]. The VCP333 or VCP102 compounds or DMSO vehicle were infused after five minutes of reperfusion for 15 min.

### 2.2. Effluent Collection for Cardiac Troponin I Analysis

Effluent samples (0.5–1 mL) were collected at selected time periods during the experimental protocol. Samples were immediately frozen at −80 °C for up to three months before analysis to preserve sample quality and prevent denaturation of proteins. Tissue cardiac troponin I (cTnI) has been shown not to deteriorate at this temperature and for this time period. An effluent control experiment was also undertaken to collect samples for comparison against pre-ischaemic samples from the ischaemia-reperfusion protocol. To investigate the levels of oncotic necrosis, (cTnI) concentrations were determined from effluent samples using the murine troponin I ELISA kit (Life Diagnostics Inc. PA, USA) according to the directions of the manufacturer.

### 2.3. Statistical Analysis

All data are expressed as mean ± SEM of the indicated numbers of determinations. Where baseline reperfusion values for LVDP, EDP, coronary flow rate and contractile function are presented, a two-way ANOVA was used. A Tukeys *post-hoc* test was used to determine the significance between the values. Cardiac troponin I samples were analysed using a one-way ANOVA. Differences were considered significant at *p* < 0.05.

## 3. Results and Discussion

### 3.1. Effect of Allosteric Modulator VCP333 Infusion during Early Reperfusion on Functional Recovery of Mouse Isolated Hearts

**Figure 2:**
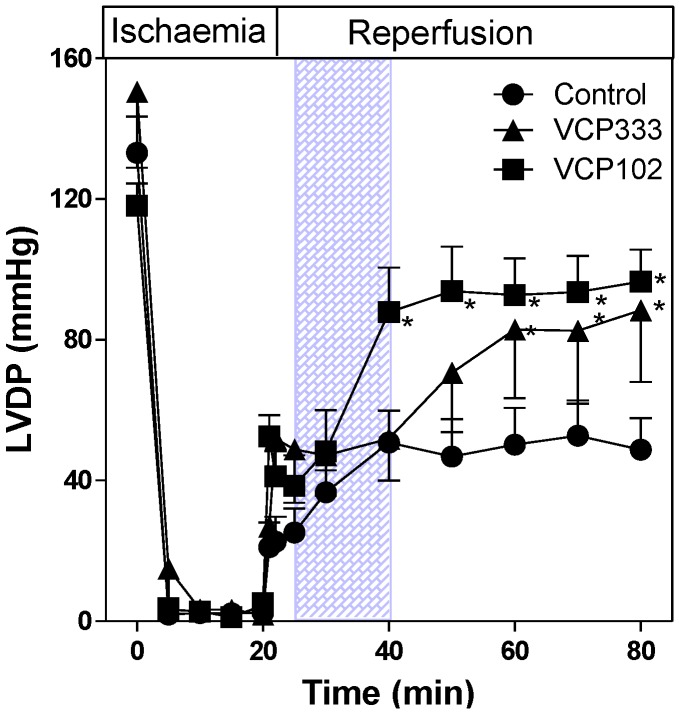
The effect of 15-min allosteric modulator VCP333 (1 μM) or VCP102 (10 μM) infusion on left ventricular developed pressure (LVDP) in mouse isolated hearts following 20-min of global ischaemia. Values are mean ± SEM, n = 6–10 per group, * *p* < 0.05 vs control.

The effect of VCP333 infusion during early reperfusion on LVDP are shown in Figure 2. Significant decreases in LVDP are shown in control hearts during ischaemia and early reperfusion (*p* < 0.05). At the end of the protocol, LVDP values in isolated control hearts recover to approximately 36% of pre-ischaemic values. In hearts treated with VCP333 (1 μM), the LVDP in hearts at the end of 60 min reperfusion recovered to ~58% of pre-ischaemic values compared to that of the control group (*p* < 0.05). Similarly, in hearts infused with VCP102, LVDP recovered to 74% of pre-ischaemic function following 20 min reperfusion and maintained this level of function until the end of 60 min reperfusion. At the end of the perfusion protocol recovery of VCP102 treated hearts was also greater than control hearts (*p* < 0.05).

**Figure 3:**
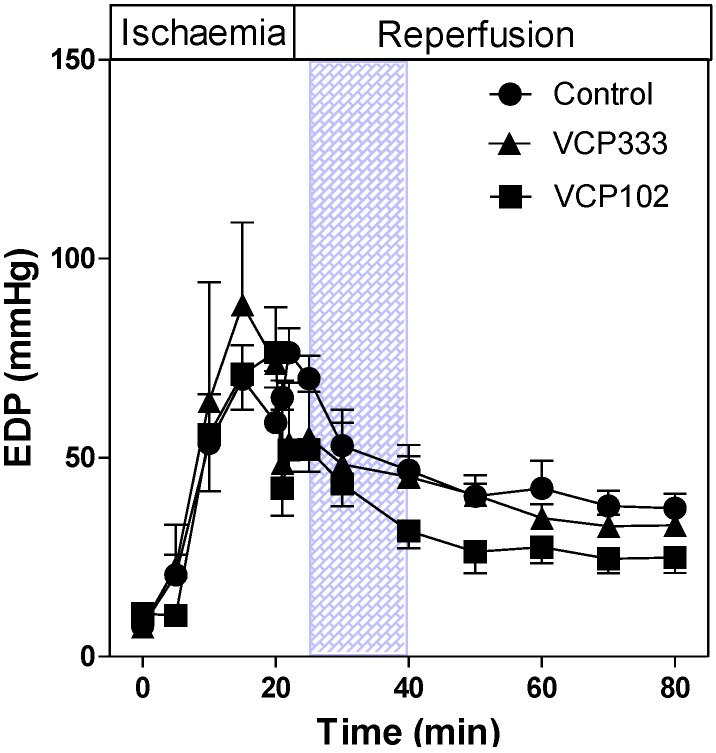
The effect of 15-min allosteric modulator VCP333 (1 µM) or VCP102 (10 µM) infusion on end diastolic pressure (EDP) in mouse isolated hearts following 20-min of global ischaemia. Values are mean ± SEM, n = 6–10 per group.

The EDP in control mouse hearts increased during 20 min ischaemia and slowly decreased during reperfusion (see Figure 3). Hearts treated with VCP102 or VCP333 during reperfusion had similar EDP values compared to control hearts throughout the ischaemia-reperfusion protocol.

**Figure 4:**
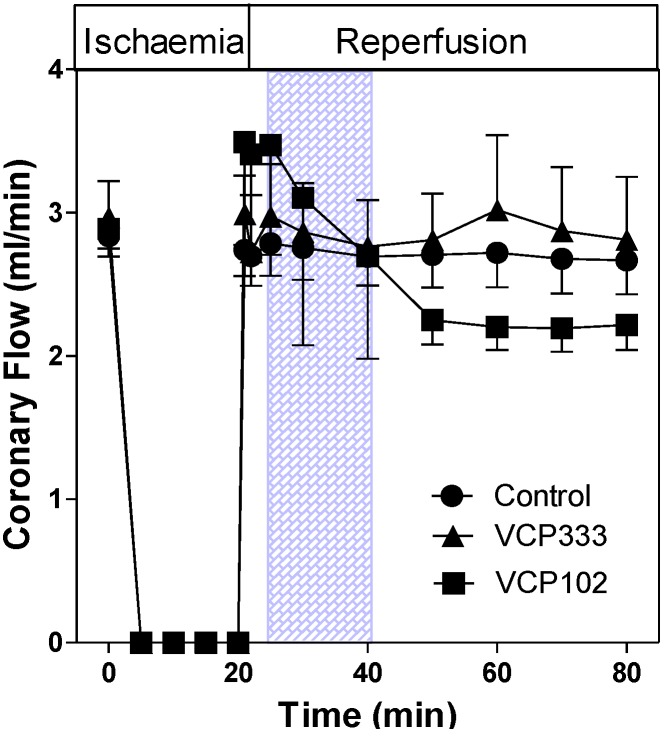
The effect of 15-min allosteric modulator VCP333 (1 μM) or VCP102 (10 μM) infusion on coronary flow (mL/min) in mouse isolated hearts following 20-min of global ischaemia. Values are mean ± SEM, n = 5–9 per group.

**Figure 5:**
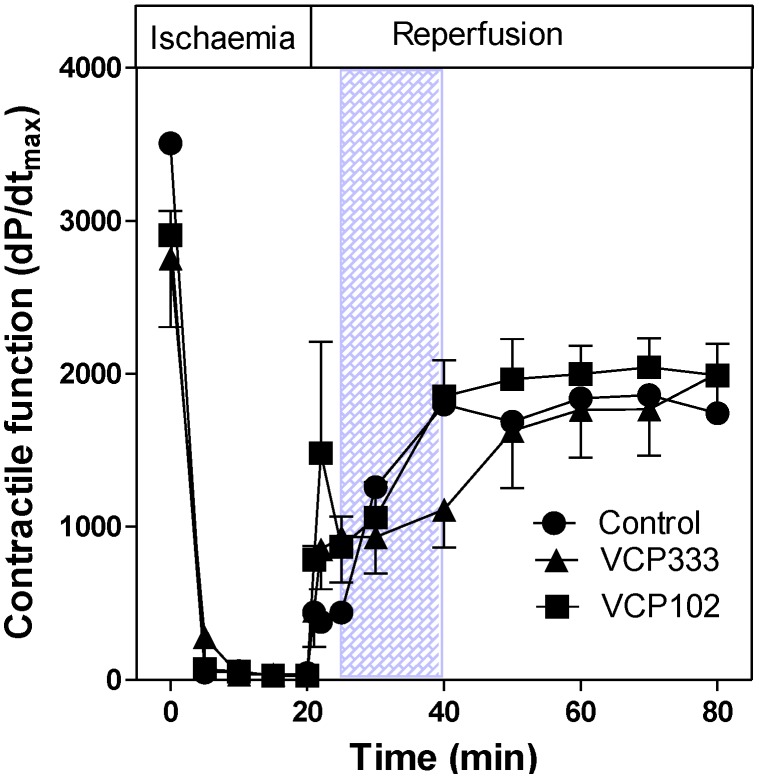
The effect of 15-min allosteric modulator VCP333 (1 μM) or VCP102 (10 μM) infusion on contractile function (dP/dt_max_) in mouse isolated hearts following 20-min of global ischaemia. Values are mean ± SEM, n = 6–10 per group.

**Figure 6:**
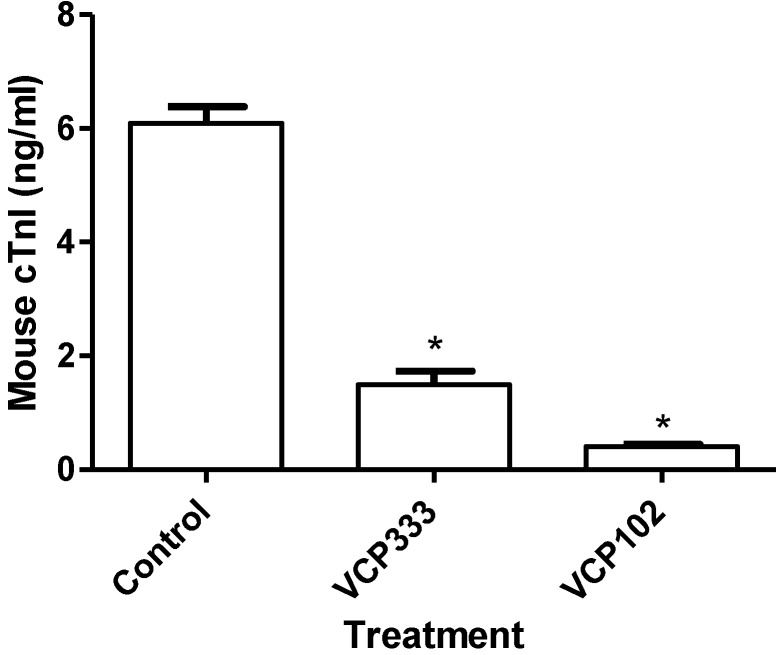
The effect of 15-min adenosine A_1_ receptor allosteric modulator VCP333 (1 μM) or VCP102 (10 µM) infusion during early reperfusion following 20-min of global ischaemia on cTnI levels. Values are mean ± SEM, * *p* < 0.05 *vs.* control.

The coronary flow rate (CRF) in isolated control hearts was approximately 2.80 ± 0.14 mL/min, n = 6) prior to ischaemia. Upon reperfusion, the CFR returned to similar flow rates as pre-ischaemic values. Post-ischaemic infusion of VCP333 or VCP102 had no effect on coronary flow rates (CFR, Figure 4). The lack of vasodilator effect of these compounds indicates that they are selective for adenosine A_1_ receptors subtypes. The heart contractile responses as measured by dP/dt_max_ (see Figure 5) returned to approximately 50% of pre-ischaemic values in control, VCP333 or VCP102 treated hearts. Drug treatment had no effect on the recovery of contractile function at any time during reperfusion in mouse isolated hearts (see Figure 6).

### 3.2. Effect of Allosteric Modulator VCP333 Infusion during Early Reperfusion on Cardiac Troponin I Production

To investigate whether the adenosine A_1_ receptor allosteric modulator VCP333 reduces the level of oncosis in cardiac tissue, cTnI levels were measured at the end of reperfusion for each group using a high-sensitivity cTnI ELISA kit. As illustrated in Figure 6, a reduction in cTnI levels was observed in tissues infused with either VCP333 or VCP102 when compared to control efflux (*p* < 0.05). The results clearly demonstrate that the control has the highest levels of oncotic cell death following 20 min ischaemia, while the infusion of the allosteric modulator VCP333 or partial agonist VCP102 significantly lower levels of cTnI in the effluent.

A large body of evidence suggests that adenosine can protect the heart against ischaemia-reperfusion injury [22,23,24]. The potential exists for novel therapeutic selection targeting allosteric sites on adenosine receptors [18,25,26]. The purpose of this study was to determine the effect of the allosteric modulator VCP333 on the functional recovery of an isolated mouse heart following an ischaemic event and to compare it to a partial agonist of the adenosine A_1_ receptor VCP102 which is known to be cardioprotective [13]. VCP333 was implemented as another approach to enhance stimulation of the adenosine A_1_ receptor following an ischaemic episode in order to take advantage of the local tissue release of adenosine following oxygen deprivation. Valant *et al.* [15] used a combination of membrane-based and intact-cell radioligand binding and multiple signalling assays to characterise the allosteric interaction between VCP333 and the adenosine A_1_ receptor.

The isolated hearts from mice were infused with VCP333 and VCP102 for 15 min during early reperfusion following 20 min of global ischaemia. Functional recovery of hearts treated with VCP333 was compared to the adenosine A_1_ receptor partial agonist VCP102.

At present the exact intracellular mechanisms and pathways triggered by allosteric binding are still not completely understood [15,16]. Evidence exists that upon binding to the receptor, allosteric ligands modulate a change in the conformation of the neighbouring orthosteric site involved in binding endogenous agonists, changing receptor affinity and/or signalling efficacy [27]. In addition, it has been proposed that allosteric enhancers may also influence functional selectivity in the signalling orthosteric ligands, therefore influencing certain intracellular signalling pathways activated by the adenosine A_1_ receptor [17].

The current results indicate the allosteric compound VCP333 may improve functional recovery in mouse hearts following an ischaemic injury. From the functional parameters investigated, only recovery of the LVDP was shown to be higher than the control group. These results may be explained by previous findings from Valant *et al.* [15], which discuss the potential bias of the allosteric modulator site and its selectivity of certain downstream signalling pathways. However, compared to the partial adenosine A_1_ receptor agonist VCP102, the effects of VCP333 were very similar with respect to recovery of LVDP. Hence, the saturable effects of the allosteric enhancer may limit stimulation of the adenosine A_1_ receptor as occurs with the use of partial agonists of the receptor.

Furthermore, there is a lack of effect of VCP333 on coronary flow rates in murine isolated hearts. The adenosine receptors responsible for coronary vasodilation in murine hearts are the A_2A_ and A_2B_ subtype [28,29]. Our research indicates that the compound VCP333 does not influence the activity of adenosine at the adenosine A_2A_ or A_2B_ receptor and showing that the compound is specific for the adenosine A_1_ receptor. It is evident from the findings that VCP333 does act to enhance the effects of local endogenous adenosine production at the adenosine A_1_ receptor in the mouse heart.

To date, there is no other research literature to directly compare the physiological effects of VCP333, however, previous studies involving other animal tissues and different allosteric compounds have been extensively investigated for the past two decades [30]. Studies involving the *in vivo* analysis of the allosteric compound 2-amino-4,5-dimethyl-3-thienyl-[3-(trifluoromethyl)phenyl]methanone (PD 81,723) in dogs, showed a functional improvement in recovery following ischaemic preconditioning [20], therefore indicating that VCP333 does have the potential of providing the heart with a specific mechanism of therapeutic cardioprotection.

In previous studies performed by our laboratory, we have shown that acute myocardial ischaemia-reperfusion injury induces apoptosis and it is this response which is attenuated by adenosine A_1_ receptor using both full and partial agonists specific to the receptor [13].

The current study has shown that the use of the allosteric modulator VCP333 may also inhibit downstream signalling cascades involved in the initiation of oncotic necrosis.

## 4. Conclusions

The current investigation provides some insight into the potential mechanisms involved in allosteric modulation of the adenosine A_1_ receptor in young mouse hearts subjected to ischaemia-reperfusion injury. We tested the hypothesis that activation of the adenosine A_1_ receptor by the allosteric modulator post ischaemia would improve functional recovery in the mouse heart. It is proposed that the binding of VCP333 to the adenosine A_1_ receptor enhances endogenous adenosine binding at the orthosteric site, as evidenced by the improvement in LVDP following an ischaemic insult. Furthermore, the intracellular pathways specifically activated by VCP333 via allosteric agonism may contribute to the inhibition of oncosis, and warrants further investigation.
